# A Consecutive 25-Week Program of Gait Training, Using the Alternating Hybrid Assistive Limb (HAL^®^) Robot and Conventional Training, and its Effects on the Walking Ability of a Patient with Chronic Thoracic Spinal Cord Injury: A Single Case Reversal Design

**DOI:** 10.3390/medicina55110746

**Published:** 2019-11-18

**Authors:** Atsushi Kanazawa, Kenichi Yoshikawa, Kazunori Koseki, Ryoko Takeuchi, Hirotaka Mutsuzaki

**Affiliations:** 1 Department of Physical Therapy, Ibaraki Prefectural University of Health Sciences Hospital 4733 Ami-machi Ami, Inashiki-gun, Ibaraki 300-0331, Japan; katouat@ami.ipu.ac.jp (A.K.); yoshikawak@ami.ipu.ac.jp (K.Y.); koseki@ami.ipu.ac.jp (K.K.); 2 Department of Orthopaedic Surgery, Ibaraki Prefectural University of Health Sciences Hospital 4733 Ami-machi Ami, Inashiki-gun, Ibaraki 300-0331, Japan; takeuchir@ipu.ac.jp; 3 Centre for Medical Sciences, Ibaraki Prefectural University of Health Sciences 4669-2 Ami-machi Ami, Inashiki-gun, Ibaraki 300-0394, Japan

**Keywords:** Hybrid Assistive Limb, long-term walking exercises, chronic thoracic cord injury, gait, walking ability

## Abstract

*Background and Objectives:* In this study, we examined the effect of a consecutive 25-week gait training program, consisting of 5-week alternating phases of Hybrid Assistive Limb (HAL)-assisted robot gait training and conventional gait training, on the walking ability of a 50-year-old man with a chronic thoracic spinal cord injury (SCI). *Materials and Methods:* Clinical features of this patient’s paraplegia were as follows: neurological level, T7; American Spinal Cord Injury Association Impairment Scale Score, C; Lower Extremity Motor Score, 20 points; Berg Balance Scale score, 15 points; and Walking Index for Spinal Cord Injury, 6 points. The patient completed a 100 m walk, under close supervision, using a walker and bilateral ankle-foot orthoses. The intervention included two phases: phase A, conventional walking practice and physical therapy for 5 weeks, and phase B, walking using the HAL robot (3 d/week, 30 min/session), combined with conventional physical therapy, for 5 weeks. A consecutive A-B-A-B-A sequence was used, with a 5-week duration for each phase. *Results:* The gait training intervention increased the maximum walking speed, cadence, and 2-min walking distance, as well as the Berg Balance and Walking Index for Spinal Cord Injury from 15 to 17 and 6 to 7, respectively. Walking speed, stride length, and cadence improved after phase A (but not B). Improved standing balance was associated with measured improvements in measured gait parameters. *Conclusion:* The walking ability of patients with a chronic SCI may be improved, over a short period by combining gait training, using HAL-assisted and conventional gait training and physical therapy.

## 1. Introduction

Patients with a spinal cord injury (SCI) present with various degrees of bilateral sensorimotor impairments. These impairments typically produce limitations in functional gait, such as asymmetric walking patterns, the need for orthoses, spasticity, decreases in step width and length, and abnormal stepping rhythm [1,2]. The Hybrid Assistive Limb^®^ (HAL, Cyberdyne, Inc., Tsukuba, Japan) is a wearable robot suit that senses a patient’s voluntary actions, such as real-time myoelectric potential, foot pressure, and joint angle, and assists hip and knee joint movement. The control mechanism of the HAL uses this information to provide person-specific assistance, as needed by the wearer, to achieve the continuous and cyclical features of normal gait. Details of the HAL control system have already been reported [3]. This improved voluntary drive of gait can improve the walking capacity of patients with a SCI.

Previous studies have reported on the acute improvement in walking capacity of patients with chronic SCIs after gait training using the HAL [4,5,6]. However, an optimal intervention program for HAL gait training has not been established for patients with a SCI. Motor learning occurs by repeatedly practicing in an environment where a task is actually performed [7]. Therefore, we considered that the change in walking function with HAL-assisted gait training can be improved by training in an actual over ground environment. Moreover, previous studies have reported that interventions alternating conventional gait training (A) and HAL-assisted gait training (B), in an A-B-A design, were effective in improving walking ability [8]. We adopted a longer A-B-A-B-A design than previous studies to evaluate possible cumulative benefits of alternating conventional gait training with HAL-assisted gait training. Therefore, our aim in this study was to evaluate the effect of a 25-week, consecutive, gait training program, which included 5 weeks of training with the HAL robot alternating with a 5-week conventional gait training program, on the walking ability of a patient with a chronic thoracic SCI.

## 2. Case Presentation

The ethics committee of Ibaraki Prefectural University of Health Sciences approved the report (approval number: 797, approval date: 27 December 2017). Written informed consent was obtained from the patient for publication and the use of accompanying images in this case report. The patient was a 50-year-old man who sustained a SCI secondary to a pyogenic spondylitis. The HAL-based gait training program was initiated approximately 12 months after the SCI. The clinical features of the paraplegia were as follows: neurological level, T7; American Spinal Cord Injury Association Impairment Scale (ASIA) Score, C; Lower Extremity Motor Score (LEMS), 20 points; Berg Balance Scale (BBS) score, 15 points; and a Walking Index for Spinal Cord Injury (WISCI-II) score of 6 points. The patient was able to walk 100 m, using a walker and bilateral ankle-foot orthoses, under close supervision.

**Figure 1:**
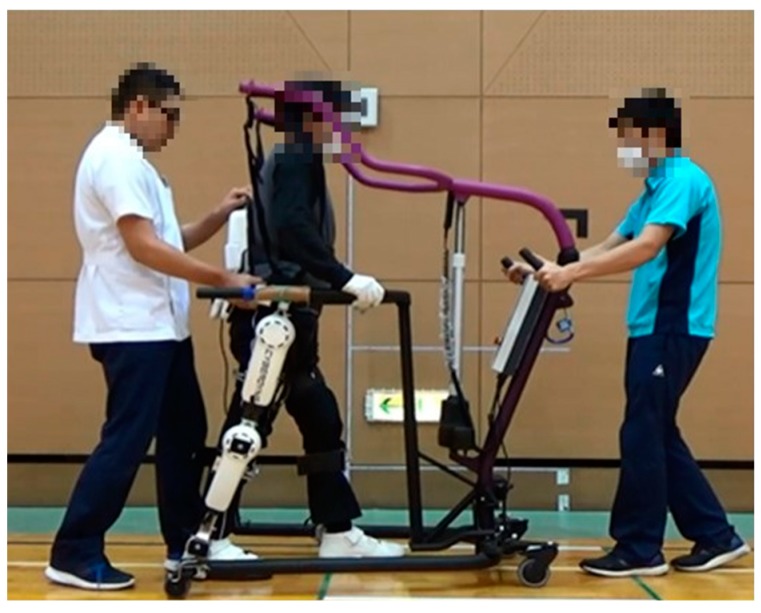
Walking exercise program using the bilateral (two leg version) Hybrid Assistive Limb^®^ (HAL) robot.

**Figure 2:**
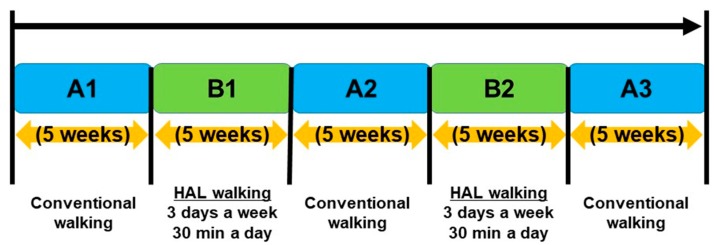
The consecutive 25-week gait training program, consisting of 5-week alternating phases of Hybrid Assistive Limb (HAL)-assisted robot gait training (B1 and B2) and conventional gait training (A1, A2 and A3).

The HAL robot used for gait training is shown in Figure 1. The intervention included two phases: (phase A) conventional gait training and physical therapy for 5 weeks, and (phase B) gait training using the HAL, combined with conventional physical therapy, for 5 weeks. Gait training using the HAL was performed 3 d/week, 30 min/session. Over the 25 weeks of gait training, a consecutive A-B-A-B-A sequence was used, with a 5-week duration for each phase (Figure 2) [9]. The bilateral version of the HAL (small size) robot was used for gait training, under the cybernic voluntary control (CVC) mode. CVC mode supports lower limb movements in response to voluntary lower limb muscle activity [10]. Specifically, under CVC mode, the HAL controller detects the bio-elective signals (BES) generated by the wearer for an intended movement and provides the torque assistance needed to perform the target movement. Normally, BESs of the antagonist and agonist muscles are used by the HAL controller, such as the quadriceps and hamstrings to control the knee. Using the hand controller, the sensitivity adjustment of the amount of assist torque according to BES can be increased or decreased with the control item “assist gain”. However, with central nervous system disorders, this assist gain can be difficult to achieve due to abnormal BES patterns, such as pathological muscle synergy (such a co-contraction of agonist and antagonists at a joint) or spasticity. In these cases, to prevent undesirable levels and/or direction of HAL-assisted motion, the BES can be filtered out, for 0% to 100%, in 10% levels. As an example, for an imbalance in level of muscle contraction or tone between antagonists and agonists, the proportion of the signal from the muscle producing stronger BES can be attenuated to provide the “assist balance” needed to yield a balanced HAL-assisted flexion/extension torque at the joint to achieve the target movement. As a result, the wearer can reproduce a motion that is close to normal. For HAL-assisted gait training, a body weight support system was used to prevent falls during the HAL gait training sessions, with no offloading of body weight during the training. The walking practice time during all interventions was limited to 30 min/d.

Phase A, consisting of conventional walking exercises and physical therapy, and phase B, walking exercises performed using the HAL robot, with conventional physical therapy, was performed for 30 min/d, 3 d/week, using A-B-A-B-A sequence. The HAL “assist gain” and “assist balance” were adjusted to yield near normal gait. This adjustment was completed within a few minutes at the start of each HAL gait training session. The conventional walking exercise was performed using parallel bars and walking aids for a maximum of 30 min. The physical therapy session included stretching, strength training, sit-to-stand training, standing balance exercises, and practicing of other movements necessary for activities of daily living.

The following gait-specific outcomes were measured 1–2 times per week: the 10 m walk time; self-selected walking speed (SWS); maximum walking speed (MWS); stride length, cadence; and the 2-min walk test (2MT). The BBS, WISCI-II, and the Modified Ashworth Scale (MAS) for knee extensor and flexor and ankle plantarflexor spasticity were measured at baseline and at the end of each phase. All physical therapy assessments were performed prior to the gait intervention, without using the HAL. A Bonferroni test was used for multiple comparisons of the mean values of measured outcomes between the A1 phase and the subsequent phases of training, as well to compare the mean between adjacent phases of training. The level of significance was set at 0.05. We used statistical software (SPSS version 24, IBM Corp, Armonk, USA) for all analyses.

**Figure 3:**
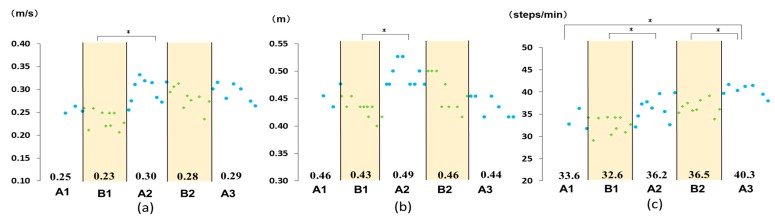
Measured outcomes: (**a**) self-selected comfortable walking speed; (**b**) stride length; and (**c**) cadence. Numbers in the graph indicate the mean value for each phase. * *P* < 0.05.

**Figure 4:**
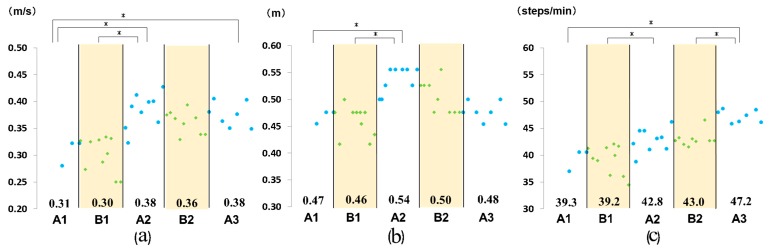
Measured outcomes: (**a**) maximum walking speed; (**b**) stride length; and (**c**) cadence. Numbers in the graph indicate the mean value for each phase. * *P* < 0.05.

The walking speed, stride length, and cadence at the SWS and MWS are summarized in Figure 3 and Figure 4, respectively. A comparison of phase A1 to all other phases revealed a significant increase in the MWS from phase A1 to A2, as well as from phase A1 to A3, an increase in stride length at MWS from phase A1 to A2, and an increase in cadence, both at MWS and SWS, from phase A1 to A3 (Figure 3 and Figure 4). Comparison of performance across adjacent phases revealed a significant increase from phase B1 to phase A2 in the SWS and MWS, from phase B1 to A2 in stride length at SWS and MWS, and from phase B1 to A2 and B2 to A3 in cadence at SWS and MWS (Figure 3 and Figure 4).

Global parameters improved overall (Table 1), including the 2MT (from 20 to 25.86 m), BBS score (from 15 to 17), standing unsupported time and standing time unsupported with feet together, and the WISCI-II score (from 6 to 7, with a progression from using a walker to elbow crutches). There was no change in the MAS (spasticity) score.

## 3. Discussion

Overall, the combined phase A and B gait training program improved functional gait capacity in our 50-year-old patient with a chronic T7 chronic SCI. MWS was significantly increased in the A2 and A3 phases, compared to the A1 phase, with these improvements in walking speed resulting from an increase in stride length and cadence.

Gait training for SCI using the HAL in previous studies that reported an improvement in gait speed was performed 5 d/week for 12 weeks [4,5,6]. In this study, the training program consisted of conventional gait training for 3 × 5 weeks in phase A and the HAL training combined with conventional gait training for 2 × 5 weeks in phase B. During the phases of HAL assistance, training was performed 3 d/week. Although the frequency and duration of gait training using the HAL were short, walking ability improved. Compared to a previous study in which HAL gait training was conducted for 12 weeks, the difference in the change in walking speed in our study was small, but the walking speed achieved was faster [6]. Therefore, if HAL training was combined with conventional gait training and physical therapy, the effective duration might be less extensive overall. The observed change in the patient’s gait pattern resulted from an underlying improvement in the stride length and cadence. Furthermore, throughout the intervention period, standing balance improved, allowing for a change in gait aid used, from a walker to elbow crutches. During HAL-assisted gait training, the level of assistance provided was determined using an algorithm that combined voluntary muscle activity and weight shift detected by the HAL. In this way, patients wearing the HAL are likely to learn that the HAL robot provides appropriate assistance when weight shifts are performed symmetrically in a timely manner. Therefore, it is possible that HAL-assisted gait training improved the base of support, balance, and lower limb symmetry associated with during gait in our patient. Previous studies reported an improvement in balance ability with HAL-assisted gait training in patients with central nervous system diseases [11], which is consistent with our findings. Improvement in the balance function and weight shifting capacity of our patient during gait likely contributed to the improvement in cadence and stride length, associated with an overall improvement in his walking pattern. Moreover, the change to using crutches indicates the ability to have achieved an alternate (right/left) gait pattern with HAL-assisted training, which we reasonably believe to be a result of the improvement in weight shifting and balance. The improvement in the WISCI-II score achieved by our patient was greater than the improvement reported on subgroup analysis (*n* = 8) in a previous study [6].

However, it is unclear if the increase in walking speed observed immediately after phase B (HAL-assisted training) was directly affected only by the HAL-assisted gait training, considering the decrease in speed during phase B. This decrease in speed could reflect fatigue and changes in lower limb spasticity associated with the use of the HAL during gait. It is possible that HAL training would cause fatigue in patients with a chronic SCI because of changes in the muscle fiber type and muscle atrophy that occur after a SCI [12]. In order to clarify this point, further research is warranted. It is necessary to analyze changes in lower limb fatigue and spasticity using electromyography in order to determine what influence HAL directly had on the walking ability of the patient with a SCI.

In interpreting our findings for practice, it is important to note that previous studies have shown that incomplete SCI lesions of the thoracic spine, including spastic motor behavior, appear to be a nonsignificant, negative predictor for training-related improvements [6]. Therefore, it will be necessary to examine long-term intervention effects in patients without SCI-associated spasticity.

## 4. Conclusions

Recognizing that a single case prevents generalization, our findings do support the potential for the gait training alternating HAL-assisted gait training and conventional gait training across consecutive phases, to improve functional gait capacity after a chronic thoracic SCI.

## Figures and Tables

**Table 1:** Measured outcomes for the 2-min walk test (2MT), Berg Balance Scale (BBS), Walking Index for Spinal Cord Injury Score (WISCI-II), and Modified Ashworth Scale (MAS).

Measured Outcomes	Pre-Intervention	A1	B1	A2	B2	A3
2MT (m)	20	20	24.56	30	24.85	25.86
WISCI-II (scores)	6	6	6	6	7	7
BBS (scores)	15	15	16	16	17	17
MAS						
Knee extension	(1+/1+)	(1+/1+)	(1+/1+)	(1+/1+)	(1+/1+)	(1+/1+)
Knee flexion	(1+/1+)	(1+/1+)	(1+/1+)	(1+/1+)	(1+/1+)	(1+/1+)
Ankle plantar flexion	(2/2)	(2/2)	(2/2)	(2/2)	(2/2)	(2/2)

2MT—2-min walk test; WISCI—Walking Index for Spinal Cord Injury; BBS—Berg Balance Scale; MAS—Modified Ashworth Scale.
